# Effects of Hydroxyethyl Cellulose and Sulfated Rice Bran Polysaccharide Coating on Quality Maintenance of Cherry Tomatoes during Cold Storage

**DOI:** 10.3390/foods12173156

**Published:** 2023-08-22

**Authors:** Guige Liu, Bingjie Chen, Hongru Liu, Xiao Wang, Yi Zhang, Cunfang Wang, Chenxia Liu, Yaoguang Zhong, Yongjin Qiao

**Affiliations:** 1 College of Food Science and Technology, Shanghai Ocean University, Shanghai 201306, China; 13761411201@163.com; 2 Institute of Crop Breeding and Cultivation, Shanghai Academy of Agricultural Science, Shanghai 201403, China; chenbingjie0204@126.com (B.C.); hear2008dream@163.com (H.L.); wangxiao.0127@163.com (X.W.); zhangyi_ppls@hotmail.com (Y.Z.); fhwcf@126.com (C.W.); liuchenxia@saas.sh.cn (C.L.)

**Keywords:** preservative coating, delay senescence, fresh-keeping

## Abstract

Cherry tomatoes are easily damaged due to their high moisture content. A composite coating was developed to delay deterioration and prolong storage by mixing antibacterial sulfated rice bran polysaccharides (SRBP) and edible hydroxyethyl cellulose (HEC) with film-forming properties. The effects of HEC, HEC-5% SRBP, and HEC-20% SRBP preservative coatings on the maintenance of the quality of cherry tomatoes (*LycopersivonesculentumMill.*, *Xiaohuang F*2) during cold storage were investigated. The HEC-20% SRBP coating significantly reduced tomato deterioration and weight loss, delayed firmness loss, decreased polyphenol oxidase activity, and increased peroxidase activity. Furthermore, cherry tomatoes treated with HEC-20% SRBP maintained high levels of titratable acid, ascorbic acid, total phenols, and carotenoids. Cherry tomatoes coated with HEC-SRBP also had higher levels of volatile substances and a greater variety of these substances compared to uncoated tomatoes. In conclusion, the HEC-20% SRBP coating effectively delayed deterioration and preserved cherry tomatoes’ nutrient and flavor qualities during postharvest cold storage, suggesting it could be a novel food preservation method.

## 1. Introduction

Cherry tomatoes are highly sought after by consumers worldwide due to their richness in sugars, organic acids, minerals, and vitamins [1,2]. These tomatoes are also a good source of vitamin C, lycopene, and beta-carotene [3]. Cherry tomatoes are listed as one of the priority fruits and vegetables by the Food and Agriculture Organization of the United Nations, as they can promote growth and development, enhance immune function, delay aging, and prevent and fight cancer [4]. However, the high water content of cherry tomatoes makes them difficult to store. After 5 to 6 days of storage at room temperature (25 °C), they lose their edible and commercial value due to water loss, crumpling, browning, and decay [5], leading to significant economic losses in the food industry.

Currently, the main methods used for cherry tomato preservation are modified atmospheres, low temperatures, and chemical preservation. However, the modified atmosphere preservation method is costly, whereas chemical preservation is associated with safety issues and environmental pollution, significantly limiting the application of these methods in fruit and vegetable preservation. Although cold storage is currently the most widely used method to store and preserve cherry tomatoes after harvesting [6], this is subject to several shortcomings, including water loss, rapid firmness decline, and mildew’s rapid growth. Hence, additional preservation techniques during low-temperature storage are often necessary to meet market demand, of which an edible coating is the most effective [7,8]. Edible film-coating technology, an eco-friendly preservation method, offers significant advantages such as low cost, simplicity in operation, wide applicability, and easy utilization, and it contributes to the reduction in non-biodegradable packaging material overuse [9]. Edible coatings create a barrier that reduces water loss, respiration rate, and migration of oxygen and other gases in fruit to delay physicochemical and biological deterioration, thus prolonging the shelf life of the fruit [10].

Hydroxyethyl cellulose (HEC) is a hydrophilic, odorless, and non-toxic cellulose derivative that is widely used in the pharmaceutical, cosmetic, and food industry due to its advantageous properties such as good film formation, degradability, and biocompatibility [11]; however, it is also associated with several shortcomings, such as poor strength, ductility, and load capacity, and it is thus necessary to use it in conjunction with other substances [12]. For example, an edible coating made of a 1:05 ratio of HEC and sodium alginate (SA), together with asparagus waste extract, extends the shelf life of strawberries up to 8 days at 25 °C and 80% relative humidity [10]. Polysaccharides, well-documented sources for developing renewable green materials, show promise in the form of rice bran polysaccharides given their abundance, low costs, and superior functional properties, such as antioxidant and antibacterial activities [13]. Previous studies have shown that introducing a sulfate group in the polysaccharide can alter the nature and structure of the polysaccharide, improving its antioxidant and antibacterial properties [14,15]. Our preliminary studies have verified these results; rice bran polysaccharides’ antibacterial and antioxidant activities were enhanced after sulfated modification. Hence, these sulfate-modified polysaccharides can be used as a natural antibacterial substance to enhance the antibacterial and preservation properties of the coating materials. To our knowledge, using HEC/SRBP-composite coatings for fruit storage has not been investigated. Therefore, this study aimed to examine the impact of HEC-SRBP coating on cherry tomatoes’ preservation under low-temperature storage conditions, intending to develop a preservation method that enhances the quality of stored cherry tomatoes.

## 2. Materials and Methods

### 2.1. Materials

Cherry tomatoes (*LycopersivonesculentumMill.*, *Xiaohuang F*2) were harvested from Fengxian Orchard (Shanghai, China) at the half-ripe stage. For experimental purposes, tomatoes of similar size and uniform maturity, free from mechanical damage, pests, and diseases, were selected.

### 2.2. SRBP

The assessment of the rice bran polysaccharide (RBP) was conducted as described by Wang et al. [16] and Li et al. [17]. Initially, degreased rice bran was extracted with water (at a ratio of 1:20, *w*/*v*) at 90 °C for 2 h and filtered twice. Subsequently, α-amylase and the Sevag reagent (n-butanol and trichloromethane 1:4, *v*/*v*) were used to remove starch and protein. The extract was centrifuged and concentrated, followed by precipitation of the concentrate overnight with ethanol. The precipitate was dissolved in water, dialyzed against distilled water, and freeze-dried to obtain RBP. In the subsequent reaction, RBP (600 mg) was mixed with dimethyl sulfoxide (90 mL), stirred at 25 °C for 1 h, and reacted with pyridine trioxide complex (20 times the mass of RBP) at 55 °C for 2 h. After cooling, 1 mol/L NaOH was added to neutralize the reaction solution, after which the material was dialyzed, concentrated, and freeze-dried to obtain SRBP.

### 2.3. Preparation of Coatings

The HEC-SRBP coating was developed using the protocol described by Akhtar et al. [18]. First, HEC solution (1 g HEC in 100 mL sterile distilled water) was stirred at 80 °C for 1 h. After cooling to room temperature, glycerol (30%, *w*/*w*) was added as a plasticizer. Different concentrations of SRBP (0, 5, 10, 15, 20, and 25% of HEC, *w*/*w*) were added to the HEC solution, and the solutions were stirred at room temperature for 0.5 h, followed by degassing under vacuum for 1 h. The coating solutions containing different concentrations of SRBP were prepared, namely, HEC, HEC-5% SRBP, HEC-10% SRBP, HEC-15% SRBP, HEC-20% SRBP, and HEC-25% SRBP.

### 2.4. Preservation of Cherry Tomatoes

The cherry tomatoes were washed, disinfected with sodium hypochlorite for 15 min (2%, *v*/*v*), and dried on paper towels. Subsequently, in groups of 150, the tomatoes were randomly macerated for 1 min in distilled water (CK), HEC, HEC-5% SRBP, HEC-10% SRBP, HEC-15% SRBP, HEC-20% SRBP, and HEC-25% SRBP and dried. The effects of different concentrations of SRBP treatments on the color and sensory characteristics of the cherry tomatoes were analyzed immediately, so as to select the appropriate SRBP concentration for subsequent storage experiments, which were conducted under the following conditions: the tomatoes were refrigerated at 4 °C for 12 days and then stored at 25 °C for 3 days.

### 2.5. Determination of Physicochemical Quality

#### 2.5.1. Color

A colorimeter (CM-5, Hangzhou Keshengxing Instrument Co., Ltd., Hangzhou, China) was used to measure the surface color (L*—luminosity, a*—redness/greenness, b*—yellowness/blueness) of the tomatoes according to the method described by Liu et al. [2]. The total color difference (ΔE*) was calculated using the following formula:

$$\Delta E=\sqrt{{\left({\Delta L}^{*}\right)}^{2}+{\left({\Delta a}^{*}\right)}^{2}+{\left({\Delta b}^{*}\right)}^{2}\;}$$

#### 2.5.2. Sensory Evaluation 

Using a hedonic scale, 20 trained judges (10 men and 10 women aged 25–40) assessed the cherry tomatoes’ color (bright yellow and uniform color), flavor (sweet and sour taste with a strong aroma), texture (moderate hardness), and overall acceptability. The scale ranged from 5 to 25 with 5 = extremely poor, 10 = poor, 15 = acceptable, 20 = very good, 25 = excellent.

#### 2.5.3. The Rate of Decay and Weight Loss 

Rotten fruit is defined as fruit that has leaked juice and has become severely softened or decayed. The decay rate and weight loss were determined using the following formulas:

$$\mathrm{weight}\;\mathrm{loss}\;(\%)=\frac{\mathrm{the}\;\mathrm{number}\;\mathrm{of}\;\mathrm{rotten}\;\mathrm{tomatoes}}{\;\mathrm{the}\;\mathrm{initial}\;\mathrm{number}\;\mathrm{of}\;\mathrm{cherry}\;\mathrm{tomato}\;}\;\times \;100$$

$$\mathrm{weight}\;\mathrm{loss}\;(\%)=\frac{\mathrm{Initial}\;\mathrm{weight}\;-\;\mathrm{Final}\;\mathrm{weight}}{\mathrm{Initial}\;\mathrm{weight}}\;\times \;100$$

#### 2.5.4. Firmness

A texture analyzer (TA-XT plus, Stable Micro Systems, Surrey, UK) was used to measure three equidistant areas on the equatorial part of the tomatoes, utilizing a p/2 probe with a speed of 2.0 mm/s and a depth of 10 mm. These measurements were conducted 12 times for each set of tests, with the results expressed in Newtons (N).

### 2.6. Nutrient Composition

#### 2.6.1. Total Soluble Solids (TSS), Titratable Acidity (TA), and Ascorbic Acid

The cherry tomato samples were filtered through two layers of gauze, and the TSS contents in the pulp were determined using a refractometer (WYA-ZT, Shanghai Yidian Physical and Optical Instruments Co., Ltd., Shanghai, China). The TA of the tomatoes was expressed as a percentage of the citric acid contents, as described by Mustapha et al. [19], while the ascorbic acid content was evaluated by the 2,6-dichlorophenol indophenol assay [20].

#### 2.6.2. Total Phenolic (TP) Content

The TP content was measured using the Folin–Ciocalteu method [21]. The sample (1 g) was homogenized in methanol (3 mL, 80% (*v*/*v*)) containing 2% formic acid and centrifuged. The supernatant (0.5 mL) was added to a solution containing a Folin–Ciocalteu reagent (1 mol/L, 1 mL) and sodium carbonate solution (3.5 mL, 1 mol/L) and reacted in a water bath at 30 °C for 1 h. Subsequently, the absorbance of the solution at 760 nm was measured, and the TP content was calculated using gallic acid as the standard.

#### 2.6.3. Carotenoids

The carotenoid contents were determined using a plant carotenoid kit (Wuhan Qinzhijie Biotechnology Co., Ltd., Wuhan, China).

$$\mathrm{Carotenoids}\;(\mathrm{mg}/\mathrm{kg})=\frac{A_{400}\;\times \;V\;\times \;D}{\varepsilon \;\times \;d\;\times \;W}\times 10^{6}$$

In this formula, A440 represents the absorbance at 440 nm, V represents the volume of the sample extracts, D stands for the dilution ratio, ε signifies the extinction coefficient of carotenoid (250 L/g/cm), d is the light diameter of a 96-well plate (0.5 cm), and W corresponds to the weight of the sample.

### 2.7. Enzyme Activity

Polyphenol oxidase (PPO) activity was assayed as described by Zeng et al. [22] with minor modifications. Cherry tomato pulp (0.5 g) was suspended in sodium phosphate buffer (0.5 mL, 0.1 M, pH 7.0) and ground into a pulp, followed by centrifugation. Subsequently, 20 µL of the supernatant was mixed with sodium phosphate buffer (200 µL, 0.1 M, pH 7.0) and catechol solution (50 µL, 0.1 M), and the absorbance was measured immediately at 420 nm. An absorbance change of 0.001 per minute represented one unit of enzyme activity.

Peroxidase (POD) activity was measured following Zeng et al.’s method [22]. The tomato pulp (0.5 g) was suspended in sodium phosphate buffer (1.5 mL, 50 mmol, pH 7.8) and centrifuged. Subsequently, 20 µL of the supernatant was added to 100 µL of 0.25% (*v*/*v*) guaiacol solution, followed by 20 µL hydrogen peroxide (0.75%, *v*/*v*) to initiate the reaction. Absorbances were read at 470 nm.

$$U=\frac{{\Delta \mathrm{OD}}_{420}\;\times \;V}{V_{s}\;\times \;m}$$

$$U=\frac{{\Delta \mathrm{OD}}_{470}\;\times \;V}{V_{s}\;\times \;m}$$

In these equations, △OD_420_ and △OD_470_ represent the changes in absorbance per minute, V stands for the total volume of sample extracts (mL), vs. refers to the liquid volume of the sample taken during the determination (mL), and m is the mass of the sample (g).

### 2.8. Malondialdehyde (MDA)

The MDA content was analyzed as described by Zeng et al. [22]. The tomato pulp (1 g) was homogenized in 5 mL of 10% (*w*/*v*) trichloroacetic acid and centrifuged. Subsequently, 2 mL of the supernatant was mixed with 2 mL of 0.6% (*w*/*v*) thiobarbituric acid, heated in boiling water for 15 min, and rapidly cooled. The absorbance of the mixture was measured at 450, 532, and 600 nm, and the MDA content was calculated as 6.45 × (A532–A600) − 0.56 × A450.

### 2.9. Volatile Substances

The contents of volatile aromatic substances in the cherry tomatoes were analyzed using solid-phase microextraction-gas chromatography-mass spectrometry (SPME-GC-MS, 7890-5975, Agilent Technologies Ltd., Palo Alto, CA, USA), following the method described by Li et al. [23] and Tsanasidou et al. [24] with minor modifications. Briefly, 5 g of cherry tomatoes (ground to powder under liquid nitrogen), 2 g of NaCl, and 0.04 mg/mL of 3-nonanone (5 μL, internal standard) were mixed in the headspace vial and vortexed. Subsequently, the mixture was extracted for 30 min using solid-phase microextraction fiber (DVB/Carboxen/PDMS, Supelco Inc., Bellefonte, PA, USA) to collect the volatile substances, followed by GC-MS analysis. A DB-WAX capillary column was used for the gas phase with high-purity helium as carrier gas at a flow rate of 1.5 m/s. The sample injection heating procedure was as follows: 40 °C held for 3 min, raised to 160 °C in 24 min, and 220 °C in 6 min. For the determination of retention indices, a mixture of n-alkanes (C5–C7 and C8–C20) was employed. The retention index (RI) for each component was calculated according to the Kovats formula:

$$\mathrm{RI}=100n+\frac{100(t_{x}\;-\;t_{n})}{t_{n+1}\;-\;t_{n}}$$

In this formula, RI represents the retention index of the analyzed component; tx represents the retention time of the measured component, min; t_n_ and t_n+1_ represent the retention time of n-alkanes with carbon number n and n + 1, respectively, min; and t_n_ < t_x_ < t_n+1_.

The content of each volatile substance (semi-quantitative) was calculated using the internal standards as a reference, with the following formula:

$${\;\mathrm{Volatile}\;\mathrm{component}\;\mathrm{content}\;(µg\cdot \mathrm{kg}}^{-1})=\frac{\;\mathrm{The}\;\mathrm{peak}\;\mathrm{area}\;\mathrm{of}\;\mathrm{each}\;\mathrm{component}\;\times \;\mathrm{Internal}\;\mathrm{standard}\;\mathrm{quality}/\mathrm{mg}}{\mathrm{Internal}\;\mathrm{standard}\;\mathrm{peak}\;\mathrm{area}\;\times \;\mathrm{Sample}\;\mathrm{quality}/g}{\;\times \;10}^{6}$$

### 2.10. Data Analysis

The Origin 2018 software was used for data visualization. SPSS 22.0 software was used to determine the significant differences. Analysis of variance (ANOVA) was used for statistical analysis of the results. *p*-values < 0.05 were considered significant, and significant differences are indicated by letters in the same column in tables.

## 3. Results

### 3.1. Effect of the Coating on the Physicochemical Quality of Cherry Tomatoes

#### 3.1.1. Color and Sensory Score

**Figure 1:**
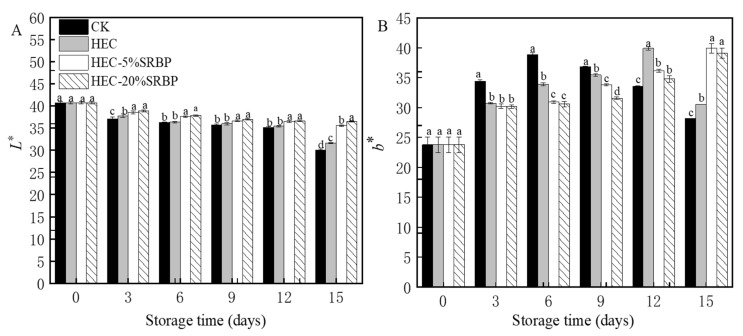
Effects of the coating on the L* (**A**) and b* (**B**) values of cherry tomatoes. Different letters (a–d) indicate a significant difference (*p* < 0.05) between treatments on the cherry tomatoes.

Color is an important characteristic and significantly affects consumer choice. The brightness of color serves as an indicator of the light-blocking properties of the coating. Specifically, a color that is too bright suggests poor light-blocking properties, while a color that is too dark could indicate suboptimal sensory properties of the fruit [25]. The CK group, which served as a blank control, had the highest sensory score. To ascertain the optimal concentration of SRBP, we compared the color and sensory characteristics of cherry tomatoes treated with varying concentrations of SRBP. Tomatoes treated with 5%, 10%, and 15% SRBP demonstrated no significant differences in L*, a*, and b* values and sensory scores (Table 1 and Table S1), and thus, to reduce costs, we used 5% SRBP as the test concentration. At an SRBP concentration of 25%, the Δ*E* value was maximal (27.63 ± 0.11), while the sensory score was minimal, which would significantly affect consumer acceptability. This may be because of the yellowish color of the polysaccharide, which has less effect on the membrane solution when the polysaccharides are added in small amounts, and has a greater effect on the membrane solution when its content reaches 25%. Therefore, in subsequent experiments, we omitted the 25% SRBP treatment and solely used the four remaining treatments (CK, HEC, HEC-5% SRBP, and HEC-20% SRBP) to investigate the impact of preservation methods on cherry tomatoes. It was found that during storage, the L* values decreased with increasing storage time, although the differences between groups only achieved significance on day 15 (Figure 1A). Moreover, the storage period and treatment duration significantly impacted the b* value. During storage, the b* values first increased and then decreased in the CK and HEC groups while they continued to increase in the HEC-5% SRBP and HEC-20% SRBP groups (Figure 1B). The rise in b* values resulted from the accelerated chlorophyll degradation and the subsequent accumulation of carotenoids [9]. In contrast, the decrease in b* value was caused by reductions in carotenoid accumulation, indicating that the composite coating treatment could delay the ripening of cherry tomatoes.

#### 3.1.2. Weight Loss, Decay Rate, and Surface Appearance

**Figure 2:**
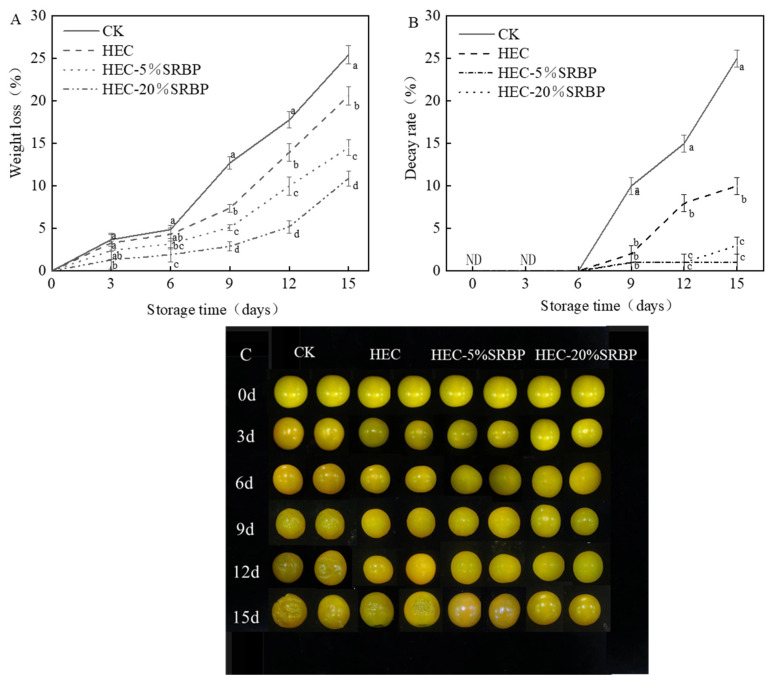
Effect of coating on weight loss (**A**), decay rate (**B**), and surface appearance (**C**) of cherry tomatoes. Different letters (a–d) indicate a significant difference (*p* < 0.05) between treatments on the cherry tomatoes.

Postharvest, fruits are susceptible to weight loss due to ongoing respiration and transpiration. This process leads to a decline in fruit plumpness, the loss of luster accompanied by wilting, and, subsequently, reduced edibility and commercial value. Therefore, the rate of weight loss is an important index for assessing the freshness of fruit [26]. In all treatment groups, the rate of weight loss increased (Figure 2A). However, no significant differences among the groups were observed during the initial six days of storage. After day 6, however, weight loss accelerated in the order of CK > HEC > HEC-5% SRBP > HEC-20% SRBP. On day 15, the HEC-20% SRBP coating group exhibited a weight loss of 10.91%, which was 2.33 and 1.89 times less than that in the CK and HEC groups, respectively, indicating that higher concentrations of HEC-SRBP coating slowed the rate of weight loss, probably due to the formation of a protective barrier by HEC-SRBP on the tomato surface, reducing water evaporation and inhibiting O_2_ and CO_2_ exchange, thus reducing nutrient loss [27]. Consistent with the results of this study, previous research has shown that applying a coating can reduce weight loss during storage, as demonstrated in peaches, mangoes, and bananas [28,29]. The decay rate of the cherry tomatoes decreased with increased storage time under all the treatment conditions (Figure 2B). The control tomatoes’ decay rate was 25% on the 15th day of storage, whereas the decay rate was significantly reduced in the HEC-SRBP coating group. Furthermore, the HEC-20% SRBP coating resulted in the best effects with consistently lower rates of decay, possibly due to the inhibition of microbial activity in the fruit resulting from the antibacterial properties of SRBP. This SRBP activity helped reduce decomposition by microorganisms, resulting in the superior quality of the fruit during storage. After 15 days of storage, sections of the tomatoes in the control group (CK) had become rotten, soft, and slightly odorous. Meanwhile, the coated fruit maintained a plumper and firmer consistency than those in the control group (Figure 2C).

#### 3.1.3. Firmness

**Figure 3:**
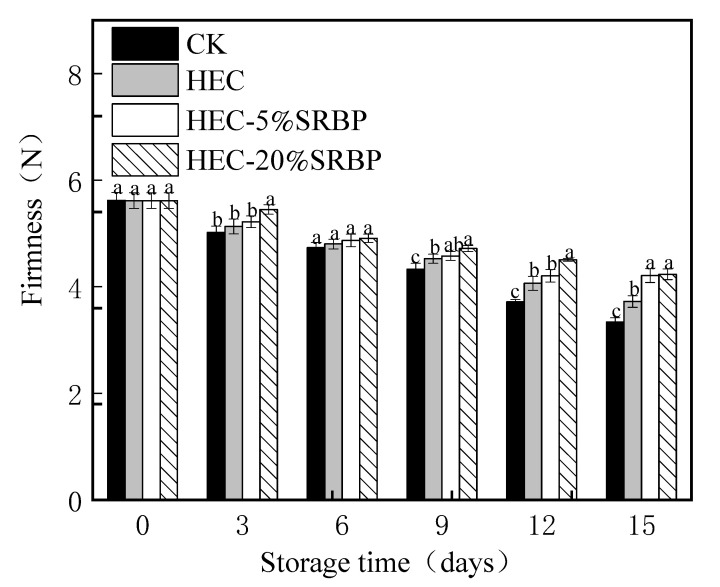
Effect of the coating on the firmness of cherry tomatoes. Different letters (a–c) indicate a significant difference (*p* < 0.05) between treatments on the cherry tomatoes.

The firmness of the cherry tomatoes decreased during the storage period (Figure 3), indicating a gradual softening of the fruit, largely due to changes in the cell wall caused by pectin degradation and the destruction of the cell structure [30]. The tomatoes in the HEC-SRBP group had higher firmness levels than those in the other groups, indicating that the HEC-SRBP coating could effectively prevent fruit from softening. This may be due to effective control of the tomato respiration, accompanied by the slowing of biochemical reactions by HEC-SRBP. These results are consistent with those of a previous study by Kim et al. [31].

### 3.2. Nutrient Composition

**Figure 4:**
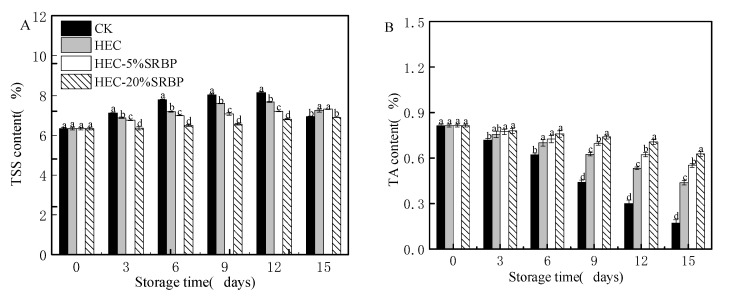

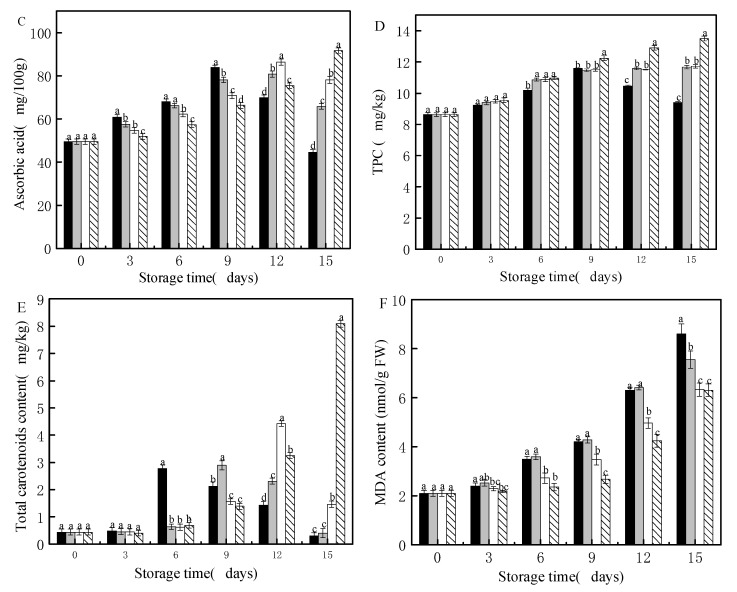
Effects of the coating on the contents of TSS (**A**), TA (**B**), Ascorbic acid (**C**), TP (**D**), Carotenoids (**E**), and MDA (**F**) of cherry tomatoes. TSS: total soluble solids, TA: titratable acidity, TP: total phenolic, MDA: Malondialdehyde. Different letters (a–d) indicate a significant difference (*p* < 0.05) between treatments on the cherry tomatoes.

The TSS content in the cherry tomatoes from the CK and HEC groups initially increased and then decreased during storage (Figure 4A). This pattern is consistent with the hydrolysis of macromolecular carbohydrates in tomatoes during the early storage stages into soluble sugars, which are continuously consumed with increased respiration and physiological activities [32]. However, the TSS content in the HEC-SRBP-coated groups showed a slow increase, probably due to inhibition of the starch conversion process and reduced respiration and metabolic activities delaying the ripening of the cherry tomatoes [33].

The TA represents an important source of adenosine triphosphate for respiration and contributes to many biochemical reactions by providing intermediate metabolites [34]. In addition, the TA is essential to maintain the taste and flavor of the fruit. The TA content in all the groups gradually decreased with storage time (Figure 4B), which may be due to the transformation of organic acids into sugars and increased respiratory energy, resulting in the degradation and consumption of more organic substances in the fruit. The TA content of the CK group decreased significantly faster than that of the HEC-SRBP-coated group, and after 15 days of storage, the TA content in the CK group was only 0.25%, while the levels in the HEC-5% SRBP and HEC-20% SRBP groups were 0.58% and 0.62%, respectively, indicating that the HEC-SRBP coating slowed the decline in TA in cherry tomatoes. Consistent with these results, Wu et al. [35] preserved cherry tomatoes with a Pullulan/oligosaccharide coating and found that the TA content in the coated group was higher than in the CK group.

Ascorbic acid serves as a cofactor in numerous enzymatic reactions. It assists in scavenging reactive oxygen radicals and inhibiting lipid peroxidation in fruit, thereby delaying processes of browning and aging [36]. Ascorbic acid is also an important nutrient in fruit and vegetables and indicates fruit freshness [20]. It was found that in all the groups except HEC-20% SRBP, the levels of ascorbic acid first increased and subsequently decreased during storage (Figure 4C). These changes in the ascorbic acid content could be attributed to its gradual increase during fruit ripening, followed by a gradual decrease due to increased physiological activities and oxidative degradation [37]. On storage day 15, the ascorbic acid contents of the HEC-5% SRBP and HEC-20% SRBP treatment groups were 1.75 and 2 times higher, respectively, than those of the CK group, suggesting a possible role of the HEC-SRBP coating in delaying the reduction in ascorbic acid content. It is possible that the formation of a relatively dense layer around the tomato by the composite coating reduced the diffusion of O_2_, delaying ascorbic acid oxidation [38].

TP plays a vital role in enhancing vegetables’ sensory and nutritional qualities [19]. We observed a consistent increase in TP content in all the coating groups; it may be that the cherry tomatoes studied were in the semi-ripe stage, and that the TP content in the cherry tomatoes showed an increasing trend with the ripening of the fruits. In addition, the coating group can effectively inhibit PPO activity and slow down the consumption rate of phenolic substances, thus maintaining a high total phenol content during storage. In contrast, the CK group exhibited an initial increase in TP content, followed by a decrease during storage (Figure 4D). This is probably due to the fact that the degradation rate of cherry tomatoes exceeded the synthesis rate during the late storage period. On days 9 and 12 of storage, the TP content in the HEC-20% SRBP treatment group was significantly higher than that in the other treatment groups (*p* < 0.05), indicating that the HEC-20% SRBP coating significantly increased the TP content of cherry tomatoes.

Carotenoids, a class of secondary metabolites, have a crucial role in plant growth and development. They can scavenge free radicals, helping prevent oxidative damage [39]. It was found that the carotenoid content of CK, HEC, HEC-5% SRBP, and HEC-20% SRBP peaked on the 6th, 9th, 12th, and 15th day (Figure 4E). At storage day 15, the carotenoid content of the HEC-20% SRBP-treated group was 8.09 mg/kg, much higher than that of the CK group (0.30 mg/kg) and the HEC group (0.41 mg/kg). We know that the accumulation of carotenoids in tomatoes is mainly concentrated in the color-turning stage and the ripening stage, and that the carotenoid content increases with the maturity of the fruit. Therefore, the result indicated that the HEC-20% SRBP treatment prevented increases in carotenoids and delayed the ripening and aging of cherry tomatoes.

### 3.3. PPO and POD Activities

**Figure 5:**
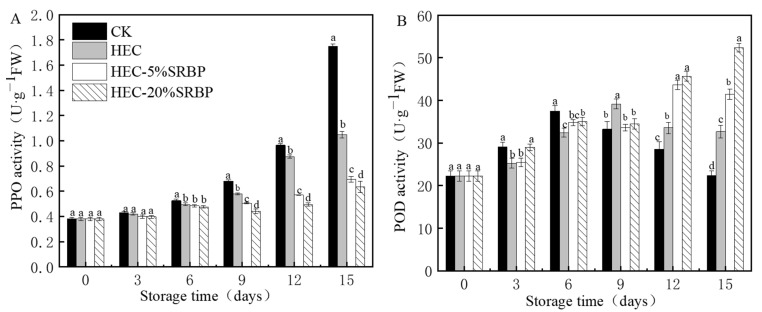
Effects of the coating on PPO (**A**) and POD (**B**) activities in cherry tomatoes. PPO: polyphenol oxidase, POD: peroxidase. Different letters (a–d) indicate a significant difference (*p* < 0.05) between treatments on the cherry tomatoes.

PPO is the principal enzyme leading to the browning of fruits and vegetables. Increased PPO activity accelerates browning by producing dark-brown melanin, affecting the quality of fruits and vegetables [40]. As shown in Figure 5A, the overall PPO activity increased in all the groups, with no significant differences among the groups during the early stages of storage; however, from days 9 to 15, the coated tomatoes had lower PPO activity, indicating that the coating delayed browning to some extent. This could be due to the reduced degradation of ascorbic acid and TP, which could inhibit PPO activity [41].

POD is an important enzyme in the plants’ reactive oxygen species-scavenging system, and variations in its activity are closely associated with fruit ripening and senescence [42]. As depicted in Figure 5B, all the treatment groups, barring the HEC-20% SRBP group, exhibited an initial increase followed by decreased POD activity. On day 15, the POD activities of the CK, HEC, HEC-5% SRBP, and HEC-20% SRBP groups were 22.41, 32.68, 41.48, and 52.39 U·g^−1^ FW, respectively. Elevated POD activity in the cherry tomatoes treated with HEC-20% SRBP led to a reduction in cellular damage and a delay in fruit ripening and senescence.

### 3.4. MDA

MDA is the primary product of membrane lipid peroxidation, and its levels indicate tissue senescence and cellular oxidation in cherry tomatoes [43]. With the increase in MDA content, the degree of membrane lipid peroxidation intensifies, the cell structure is destroyed, and the rate of senescence and cell death is accelerated. MDA levels consistently increased throughout storage (Figure 4F), indicating ongoing oxidative tissue damage. This may be due to the disruption of the dynamic balance of reactive oxygen radicals during storage as the nutrients are consumed by respiration, transpiration, and other metabolic activities of the substrates. And with the accumulation of oxidation products, the intracellular enzymatic reactions are accelerated, causing membrane lipid peroxidation and an increase in MDA content. The MDA content of the HEC-20% SRBP-composite-coated group was lowest on day 15 of storage (6.31 nmol/g FW) compared with those of other groups, indicating that the HEC-20% SRBP treatment significantly reduced membrane damage in cherry tomatoes and delayed fruit senescence. Similar to the results of this study, Zeng et al. [22] confirmed that at the end of storage, the MDA content of cherry tomatoes treated with 2.5 mg/L exogenous arachidonic acid was 6.9 nmol/g FW. In summary, HEC-20% SRBP coating increased the POD activity of cherry tomatoes, effectively eliminated reactive oxygen species, and slowed down accumulation of MDA.

### 3.5. Volatile Substances

**Figure 6:**
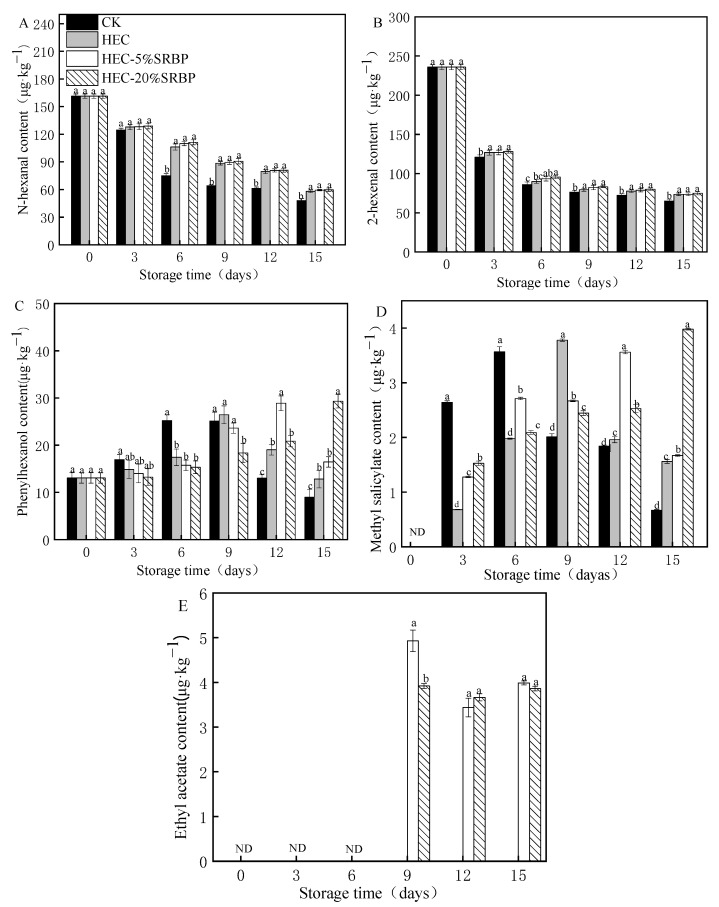
Effects of the coating on the contents of N-hexanal (**A**), 2-hexenal (**B**), phenylethyl alcohol (**C**), methyl salicylate (**D**), and ethyl acetate (**E**) in cherry tomatoes. Different letters (a–d) indicate a significant difference (*p* < 0.05) between treatments on the cherry tomatoes.

In total, 36 major compounds were detected, including 9 aldehydes, 7 alcohols, 1 ketone, 2 esters, 10 hydrocarbons, and 6 other compounds (Table S2 and Figure S1). Among the volatile compounds responsible for flavor, aldehydes exhibited the highest concentration, followed by alcohols. The retention indices of the volatiles were calculated based on retention times of the alkanes (Table S3) and also compared with the literature [44]. The retention indices of Hexanal, (E)-2-hexenal, Phenethyl alcohol, and Methyl salicylate were found to be 1079, 1219, 1928, and 1749 (Table 2), which are in agreement with those of the literature, which are 1084, 1220, 1925, and 1745, and proved that the volatiles were identified efficiently. N-hexanal and 2-hexenal, known as “green” compounds with a “grassy taste”, are known to inhibit fruit deterioration [45]. The contents of both compounds decreased gradually with the increase in storage time (Figure 6A,B). At the end of the storage period, the HEC-20% SRBP group had significantly higher contents of these substances than those of the other groups, indicating that fruits treated with a composite coating retained more volatile substances, contributing to the maintenance of freshness in the tomatoes. Alcohols play an important role in enhancing the flavor of cherry tomatoes. Phenylethyl alcohol, formed via the amino acid metabolic pathway, imparts a floral aroma to cherry tomatoes [46]; the content of phenylethyl alcohol in the CK, HEC, HEC-5% SRBP, and HEC-20% SRBP groups first increased and subsequently decreased during the storage time, reaching peaks on the 6th, 9th, 12th, and 15th days, respectively, with a ranking of peak size being HEC-20% SRBP > HEC-5% SRBP > HEC > CK (Figure 6C). These results indicated that the film coating could delay the aging of cherry tomatoes and enhance their fragrance. Methyl salicylate is associated with a “minty” flavor. The methyl salicylate content in the cherry tomatoes varied as the storage was extended (Figure 6D). By the end of the storage period, the HEC-20% SRBP group exhibited significantly higher contents of these substances than the other groups. Ethyl acetate provides a fruit-like aroma and was only found in the HEC-5% SRBP and HEC-20% SRBP groups (Figure 6E). This finding suggests that fruits treated with a composite coating preserved more volatile substances, thus contributing to freshness retention in the tomatoes. Overall, the results suggested that the HEC-20% SRBP coating could delay the ripening of cherry tomatoes, reduce the loss of volatile substances, and play a role in preserving the freshness of the fruit.

## 4. Discussion and Conclusions

We evaluated the effects of an HEC-SRBP coating film on preservation of cherry tomatoes under low-temperature conditions. Compared to uncoated and HEC-coated fruit, cherry tomatoes treated with an HEC-SRBP coating demonstrated reduced weight loss, a slower decay rate, and better maintained firmness. This coating also decreased MDA accumulation, inhibited PPO activity, enhanced POD activity, and preserved high ascorbic acid, TP, and carotenoid concentrations. Furthermore, HEC-SRBP-coated cherry tomatoes contained higher and more diverse volatile substances than uncoated tomatoes. Among all HEC-SRBP coatings, HEC-20% SRBP is the best coating method due to its effectiveness in delaying the deterioration of cherry tomatoes and preserving their nutritional and flavor qualities. These favorable results could be due to the antibacterial properties of SRBP. The synergistic interaction between HEC and SRBP forms a more robust barrier than either component alone, mitigating water evaporation and gaseous exchange, thus providing optimal storage conditions. Several researchers are currently focusing on this by taking a number of approaches to improve the film properties of HEC. For example, cellulose nanocrystals benefit the dispersion of the crosslinked HEC, which increases the elongation at break and maintains excellent tensile strength [47]. Meanwhile, Huang et al. [48] found that aramid nanofiber additives can give the HEC film excellent ultraviolet (UV) shielding and visible light transmittance. Adding carboxymethyl chitosan and ZnO enhanced the solvent resistance and UV-shielding ability and inhibited the activities of the pathogenic bacteria *Listeria monocytogenes* and *Pseudomonas aeruginosa* [12]. These enhanced properties of HEC are advantageous in food preservation applications. Nonetheless, HEC-based films have seldom been employed in fruit and vegetable preservation, underscoring the need to further explore and apply these materials. Indeed, other coating techniques used to preserve cherry tomatoes have shown beneficial results. These include carboxymethyl cellulose films that were found to improve the preservation during storage of cherry tomatoes over 15 days, maintaining almost constant fruit weight and firmness [49]. Meanwhile, edible konjac glucomannan/curdlan coatings significantly reduced weight loss and decay, delaying the decreases in the firmness and contents of cherry tomatoes’ soluble solids, total acid, and ascorbic acid [34]. Applying exogenous methyl jasmonate/gliadin-casein nanoparticles could also delay fruit ripening, maintain fruit quality, and reduce chilling injury symptoms in cherry tomatoes [50]. Thus, edible coatings have become an important method for ensuring both the safe and green preservation of cherry tomatoes. In conclusion, the HEC-SRBP coating is a novel method for maintaining the quality of cherry tomatoes during storage, providing both good application value and economic benefits.

## Figures and Tables

**Table 1:** Effects of coating on the color and sensory score of cherry tomatoes.

	L*	a*	b*	ΔE*	Sensory Score
CK	35.55 ± 1.21 ^c^	2.84 ± 1.33 ^b^	23.77 ± 1.27 ^e^	——	95 ± 2 ^a^
HEC	35.55 ± 1.21 ^c^	3.11 ± 1.03 ^b^	29.72 ± 1.01 ^d^	8.58 ± 0.18 ^f^	93 ± 1 ^a^
HEC-5% SRBP	38.64 ± 1.15 ^b^	3.32 ± 1.14 ^b^	32.56 ± 1.23 ^c^	9.33 ± 0.02 ^e^	92 ± 3 ^a^
HEC-10% SRBP	38.72 ± 1.13 ^b^	3.33 ± 1.18 ^b^	33.84 ± 1.15 ^c^	10.52 ± 0.10 ^d^	92 ± 1 ^a^
HEC-15% SRBP	38.85 ± 1.09 ^b^	3.34 ± 1.12 ^b^	34.16 ± 1.11 ^c^	10.79 ± 0.13 ^c^	91 ± 3 ^a^
HEC-20% SRBP	35.31 ± 1.14 ^c^	3.36 ± 1.17 ^b^	39.18 ± 1.03 ^b^	16.89 ± 0.24 ^b^	91 ± 2 ^a^
HEC-25% SRBP	32.26 ± 1.16 ^d^	6.39 ± 1.19 ^a^	49.48 ± 1.38 ^a^	27.63 ± 0.11 ^a^	71 ± 3 ^b^

L*: luminosity, a*: redness/greenness, b*: yellowness/blueness, ΔE*: total color difference, HEC: Hydroxyethyl cellulose, HEC-SRBP: Hydroxyethyl cellulose-sulfated rice bran polysaccharide. Different letters (a–f) indicate a significant difference (*p* < 0.05) between treatments on the cherry tomatoes.

**Table 2:** The analysis of volatile substances of cherry tomatoes.

Number	Retention Time/min	Aroma Component	RI	RI (Literature) [46]
1	13.234	n-Hexanal	1079	1084
2	19.224	(E)-2-hexenal	1219	1220
3	40.67	Phenethyl alcohol	1928	1925
4	35.65	Methyl salicylate	1749	1745

## Data Availability

Data are contained within the article. For more detailed results of the sensory evaluation and volatile substances of cherry tomatoes, see Tables S1 and S2.

## Supplementary Materials

The following supporting information can be downloaded at: https://www.mdpi.com/article/10.3390/foods12173156/s1. Figure S1: Chromatograms of volatiles substances at different times for different treatments. Table S1: Sensory evaluation of cherry tomatoes, Table S2: Effect of different treatments on volatile substances of cherry tomatoes, Table S3: The retention time of n-alkanes.

## Abbreviations

**Abbreviation**	**Name**
SRBP	Sulfated rice bran polysaccharides
HEC	Hydroxyethyl cellulose
*L**	Luminosity
*a**	Redness/greenness,
*b**	Yellowness/blueness
TSS	Total soluble solids
TA	Titratable acidity
TP	Total phenolic
PPO	Polyphenol oxidase
POD	Peroxidase
MDA	Malondialdehyde
