# Supplementary material for: Effects of Hydroxyethyl Cellulose and Sulfated Rice Bran Polysaccharide Coating on Quality Maintenance of Cherry Tomatoes during Cold Storage
Source: Foods. 2023 Aug 22;12(17):3156. doi: 10.3390/foods12173156 (PMC10486926; doi:10.3390/foods12173156)
Supplement: Supplementary file 1 [file foods-12-03156-s001.zip › foods-2459561-supplementary.pdf]

Table S1. Sensory evaluation of cherry tomatoes

| | **color** | **flavour** | **mouthfeel texture** | **overall acceptability** |
|---|---|---|---|---|
| CK | 24.5±0.6 | 23.5±0.5 | 24.0±0.4 | 23.0±0.6 |
| HEC | 23.5±0.3 | 23.5±0.2 | 24.0±0.2 | 22.0±0.3 |
| HEC-5%SRBP | 22.5±0.8 | 23.5±0.7 | 24.0±0.7 | 22.0±0.8 |
| HEC-10%SRBP | 22.5±0.3 | 23.5±0.2 | 24.0±0.2 | 22.0±0.3 |
| HEC-15%SRBP | 22.0±0.8 | 23.5±0.7 | 24.0±0.7 | 21.5±0.8 |
| HEC-20%SRBP | 22.0±0.6 | 23.5±0.4 | 24.0±0.4 | 21.5±0.6 |
| HEC-25%SRBP | 10.5±0.8 | 22.0±0.7 | 21.5±0.7 | 17.0±0.8 |

Table S2. Effect of different treatments on volatile substances of cherry tomatoes

| Type of compound | Retention time /min | Aroma component | Storage time /d | content /μg·kg-1 | | | |
|---|---|---|---|---|---|---|---|
| | | | | CK | HEC | HEC-5%SRBP | HEC-20%SRBP |
| Aldehydes | 17.53 | heptanal | 0 | $2.13^{a}$ | $2.13^{a}$ | $2.13^{a}$ | $2.13^{a}$ |
| | | | 3 | $2.31^{a}$ | $2.30^{a}$ | $2.17^{b}$ | $2.16^{b}$ |
| | | | 6 | $5.39^{d}$ | $2.70^{a}$ | $3.35^{c}$ | $2.74^{b}$ |
| | | | 9 | $4.39^{c}$ | $5.61^{d}$ | $3.88^{b}$ | $3.47^{a}$ |
| | | | 12 | $3.22^{a}$ | $3.85^{b}$ | $6.01^{d}$ | $4.55^{c}$ |
| | | | 15 | $2.10^{a}$ | $3.44^{b}$ | $4.69^{c}$ | $6.23^{d}$ |
| | 23.84 | (E)-2- heptenal | 0 | —— | —— | —— | —— |
| | | | 3 | $1.82^{c}$ | $1.77^{b}$ | $1.76^{b}$ | $1.46^{a}$ |
| | | | 6 | $8.83^{d}$ | $5.47^{c}$ | $4.83^{b}$ | $4.01^{a}$ |
| | | | 9 | $8.20^{c}$ | $8.96^{d}$ | $6.94^{a}$ | $7.20^{b}$ |
| | | | 12 | $6.52^{a}$ | $6.84^{b}$ | $9.27^{d}$ | $8.43^{c}$ |
| | | | 15 | $4.30^{a}$ | $5.55^{b}$ | $5.72^{c}$ | $9.54^{d}$ |
| | 19.224 | (E)-2- hexenal | 0 | $235.87^{a}$ | $235.87^{a}$ | $235.87^{a}$ | 235.87a |
| | | | 3 | $121.04^{a}$ | $126.74^{b}$ | $126.95^{b}$ | $128.09^{c}$ |
| | | | 6 | $85.90^{a}$ | $89.83^{b}$ | $93.34^{c}$ | $95.73^{d}$ |
| | | | 9 | $76.21^{a}$ | $79.46^{b}$ | $82.33^{c}$ | $83.15^{d}$ |
| | | | 12 | $72.26^{a}$ | $77.73^{b}$ | $78.76^{b}$ | $80.09^{c}$ |
| | | | 15 | $65.11^{a}$ | $73.52^{b}$ | $73.53^{b}$ | $74.90^{d}$ |
| | 13.234 | n-Hexanal | 0 | $161.4^{a}$ | $161.42^{a}$ | $161.42^{a}$ | $161.42^{a}$ |
| | | | 3 | $124.65^{a}$ | $127.84^{b}$ | $128.27^{b}$ | $128.91^{b}$ |
| | | | 6 | $75.06^{a}$ | $106.25^{b}$ | $109.91^{c}$ | $111.26^{d}$ |
| | | | 9 | $64.19^{a}$ | $88.56^{b}$ | $89.27^{c}$ | $90.78^{d}$ |
| | | | 12 | $61.27^{a}$ | $79.534^{b}$ | $80.82^{c}$ | $81.05^{d}$ |
| | | | 15 | $54.15^{a}$ | $58.05^{b}$ | $59.41^{c}$ | $59.84^{c}$ |
| | 26.53 | Nonanal | 0 | $2.92^{a}$ | $2.92^{a}$ | $2.92^{a}$ | $2.92^{a}$ |
| | | | 3 | $6.50^{d}$ | $4.91^{c}$ | $4.48^{b}$ | $3.39^{a}$ |
| | | | 6 | $7.99^{d}$ | $5.72^{c}$ | $5.68^{b}$ | $3.90^{a}$ |
| | | | 9 | $7.97^{c}$ | $8.04^{d}$ | $5.83^{b}$ | $5.04^{a}$ |
| | | | 12 | $7.29^{c}$ | $6.33^{b}$ | $8.23^{d}$ | $6.00^{a}$ |

| | | | | | | | |
|---|---|---|---|---|---|---|---|
| | | | 15 | 4.69[a] | 5.05[b] | 5.49[c] | 7.28d |
| | 38.44 | Trans-2,4-Decadienal | 0 | —— | —— | —— | —— |
| | | | 3 | —— | —— | —— | —— |
| | | | 6 | —— | —— | —— | —— |
| | | | 9 | —— | —— | —— | —— |
| | | | 12 | —— | —— | —— | —— |
| | | | 15 | 3.36[b] | 2.88[a] | 2.98[a] | 2.86[a] |
| | 30.92 | Benzaldehyde | 0 | 28.44[a] | 28.44[a] | 28.44[a] | 28.44[a] |
| | | | 3 | 13.65[d] | 12.81[c] | 12.33[b] | 10.81[a] |
| | | | 6 | 10.49[b] | 10.32[b] | 8.50[a] | 8.40[a] |
| | | | 9 | 9.39[d] | 7.98[b] | 8.49[c] | 7.75[a] |
| | | | 12 | 8.77[d] | 7.55[b] | 8.41[c] | 7.34[a] |
| | | | 15 | 7.85[d] | 6.23[b] | 6.93[c] | 5.82[a] |
| Alcohols | 26.29 | cis-3-Hexenol | 0 | 29.28[a] | 29.28[a] | 29.28[a] | 29.28[a] |
| | | | 3 | 23.19[a] | 24.88[a] | 24.61[a] | 24.22[a] |
| | | | 6 | 22.56[a] | 23.50[a] | 23.11[a] | 22.75[a] |
| | | | 9 | 22.13[a] | 22.51[a] | 22.70[a] | 22.96[a] |
| | | | 12 | 21.39[a] | 22.03[a] | 22.15[a] | 22.25[a] |
| | | | 15 | 10.18[a] | 15.18[b] | 16.64[c] | 17.25[d] |
| | 18.81 | 2-Methyl-1-butanol | 0 | 0.77[a] | 0.77[a] | 0.77[a] | 0.77[a] |
| | | | 3 | 1.12[a] | 0.98[a] | 0.87[a] | 0.81[a] |
| | | | 6 | 1.88[a] | 1.76[a] | 1.72[a] | 1.58[a] |
| | | | 9 | 3.56[a] | 3.47[a] | 3.32[a] | 3.19[a] |
| | | | 12 | 4.84[a] | 4.74[a] | 4.65[a] | 4.57[a] |
| | | | 15 | 7.54c | 7.24[b] | 4.68[a] | 4.65[a] |
| | 40.67 | Phenethyl alcohol | 0 | 13.06[a] | 13.06[a] | 13.06[a] | 13.06[a] |
| | | | 3 | 16.91[d] | 14.85[c] | 14.01[b] | 13.23[a] |
| | | | 6 | 25.20[d] | 17.43[c] | 15.73[b] | 15.32[a] |
| | | | 9 | 25.13[c] | 26.43[d] | 23.61[b] | 18.34[a] |
| | | | 12 | 13.01[a] | 19.03[b] | 28.88[d] | 20.84[c] |
| | | | 15 | 8.96[a] | 12.81[b] | 16.45[c] | 29.31[d] |
| | 20.74 | 1-Pentanol | 0 | 3.43[a] | 3.43[a] | 3.43[a] | 3.43[a] |
| | | | 3 | 2.61[a] | 2.52[a] | 2.59[a] | 2.61[a] |
| | | | 6 | 2.68[a] | 2.61[a] | 2.68[a] | 2.58[a] |

| | | | | | | | |
|---|---|---|---|---|---|---|---|
| | | | 9 | 3.50 $^{a}$ | 3.56$^{a}$ | 3.59$^{a}$ | 3.54$^{a}$ |
| | | | 12 | 2.96 $^{a}$ | 2.64 $^{a}$ | 2.87$^{a}$ | 3.01$^{a}$ |
| | | | 15 | 2.56 $^{a}$ | 2.55 $^{a}$ | 2.63$^{b}$ | 2.74$^{c}$ |
| | 28.51 | 1-Octen-3-ol | 0 | 11.72$^{a}$ | 11.72$^{a}$ | 11.72$^{a}$ | 11.72$^{a}$ |
| | | | 3 | 10.41$^{c}$ | 9.30$^{a}$ | 9.23$^{a}$ | 9.94$^{b}$ |
| | | | 6 | 8.28$^{a}$ | 8.69$^{c}$ | 8.38$^{b}$ | 9.53$^{d}$ |
| | | | 9 | 8.31$^{c}$ | 7.24$^{a}$ | 8.14$^{b}$ | 9.60$^{d}$ |
| | | | 12 | 8.86$^{d}$ | 6.69$^{a}$ | 6.87$^{b}$ | 7.87$^{c}$ |
| | | | 15 | 6.28$^{a}$ | 6.93$^{b}$ | 7.26$^{c}$ | 7.22$^{d}$ |
| Ketones | 24.38 | Methylheptenone | 0 | 2.35$^{a}$ | 2.35$^{a}$ | 2.35$^{a}$ | 2.35$^{a}$ |
| | | | 3 | 3.35$^{d}$ | 2.88$^{c}$ | 2.46$^{b}$ | 2.24$^{a}$ |
| | | | 6 | 3.64$^{d}$ | 3.48$^{c}$ | 3.23$^{b}$ | 2.32$^{a}$ |
| | | | 9 | 2.54$^{a}$ | 4.86$^{d}$ | 3.81$^{c}$ | 3.75$^{b}$ |
| | | | 12 | 2.02$^{a}$ | 2.49$^{b}$ | 4.93$^{d}$ | 4.17$^{c}$ |
| | | | 15 | 1.88$^{a}$ | 2.21$^{b}$ | 2.48$^{c}$ | 5.61$^{d}$ |
| Hydrocarbons | 20.76 | Sulforaphane | 0 | —— | —— | —— | —— |
| | | | 3 | 3.42$^{a}$ | —— | —— | —— |
| | | | 6 | 3.42$^{a}$ | —— | —— | —— |
| | | | 9 | 3.31$^{a}$ | 3.48$^{b}$ | —— | —— |
| | | | 12 | 3.29$^{a}$ | 3.47$^{b}$ | 3.51$^{b}$ | —— |
| | | | 15 | 3.3$^{a}$ | 3.45$^{b}$ | 3.52$^{c}$ | 3.55$^{c}$ |
| | 20.40 | $\alpha$-Terpinene | 0 | —— | —— | —— | —— |
| | | | 3 | —— | —— | —— | —— |
| | | | 6 | 3.42$^{a}$ | —— | —— | —— |
| | | | 9 | 3.31$^{a}$ | 3.48$^{a}$ | —— | —— |
| | | | 12 | 3.29$^{a}$ | 3.47$^{b}$ | 3.51$^{b}$ | —— |
| | | | 15 | 3.3$^{a}$ | 3.45$^{a}$ | 3.52$^{b}$ | 3.55$^{b}$ |
| | 5.175 | 2-Methylheptane | 0 | 1.24$^{a}$ | 1.23$^{a}$ | 1.24$^{a}$ | 1.24$^{a}$ |
| | | | 3 | 1.23a | 1.22a | 1.26a | 1.25a |
| | | | 6 | 1.24$^{a}$ | 1.24a | 1.27$^{a}$ | 1.24$^{a}$ |
| | | | 9 | 1.23$^{a}$ | 1.29$^{a}$ | 1.24$^{a}$ | 1.21$^{a}$ |
| | | | 12 | 1.25$^{a}$ | 1.27$^{a}$ | 1.26$^{a}$ | 1.26$^{a}$ |
| | | | 15 | 1.26$^{a}$ | 1.28$^{a}$ | 1.27$^{a}$ | 1.26$^{a}$ |
| | 5.625 | n-octane | 0 | 1.56$^{a}$ | 1.56$^{a}$ | 1.56$^{a}$ | 1.56$^{a}$ |

| | | | | | | | |
|---|---|---|---|---|---|---|---|
| | | | 3 | $1.44^{b}$ | $1.47^{b}$ | $1.25^{a}$ | $1.30^{a}$ |
| | | | 6 | $1.51^{a}$ | $1.54^{a}$ | $1.76^{b}$ | $1.56^{a}$ |
| | | | 9 | $1.61^{a}$ | $1.60^{a}$ | $1.69^{a}$ | $1.58^{a}$ |
| | | | 12 | $1.58^{a}$ | $1.62^{b}$ | $1.74^{b}$ | $1.59^{a}$ |
| | | | 15 | $1.49^{a}$ | $1.30^{a}$ | $1.56^{a}$ | $1.40^{a}$ |
| | 5.663 | 2,3,5-Trimethylhexane | 0 | $1.21^{a}$ | $1.21^{a}$ | $1.21^{a}$ | $1.21^{a}$ |
| | | | 3 | $1.18^{a}$ | $1.19^{a}$ | $1.17^{a}$ | $1.18^{a}$ |
| | | | 6 | $1.24^{a}$ | $1.23^{a}$ | $1.22^{a}$ | $1.26^{a}$ |
| | | | 9 | $1.24^{a}$ | $1.25^{a}$ | $1.28^{a}$ | $1.25^{a}$ |
| | | | 12 | $1.32^{a}$ | $1.33^{a}$ | $1.29^{a}$ | $1.30^{a}$ |
| | | | 15 | $1.31^{a}$ | $1.29^{a}$ | $1.27^{a}$ | $1.29^{a}$ |
| | 8.792 | 2-Methylnonane | 0 | $2.69^{a}$ | $2.69^{a}$ | $2.69^{a}$ | $2.69^{a}$ |
| | | | 3 | $2.64^{a}$ | $2.66^{a}$ | $2.65^{a}$ | $2.66^{a}$ |
| | | | 6 | $2.59^{a}$ | $2.61^{a}$ | $2.65^{a}$ | $2.64^{a}$ |
| | | | 9 | $2.56^{a}$ | $2.6^{a}$ | $2.62^{a}$ | $2.63^{a}$ |
| | | | 12 | $2.59^{a}$ | $2.61^{a}$ | $2.65^{a}$ | $2.64^{a}$ |
| | | | 15 | $2.62^{a}$ | $2.63^{a}$ | $2.64^{a}$ | $2.62^{a}$ |
| Esters | 36.65 | Methyl salicylate | 0 | —— | —— | —— | —— |
| | | | 3 | $2.64^{d}$ | $0.68^{a}$ | $1.27^{b}$ | $1.52^{c}$ |
| | | | 6 | $3.56^{d}$ | $1.98^{a}$ | $2.71^{c}$ | $2.09^{b}$ |
| | | | 9 | $2.01^{a}$ | $3.78^{d}$ | $2.67^{c}$ | $2.44^{b}$ |
| | | | 12 | $1.84^{a}$ | $1.96^{b}$ | $3.56^{d}$ | $2.52^{c}$ |
| | | | 15 | $0.6^{7a}$ | $1.56^{b}$ | $1.67^{c}$ | $3.98^{d}$ |
| | 7.09 | Ethyl acetate | 0 | $0^{a}$ | $0^{a}$ | $0^{a}$ | $0^{a}$ |
| | | | 3 | $0^{a}$ | $0^{a}$ | $0^{a}$ | $0^{a}$ |
| | | | 6 | $0^{a}$ | $0^{a}$ | $0^{a}$ | $0^{a}$ |
| | | | 9 | $0^{a}$ | $0^{a}$ | $4.93^{b}$ | $3.91^{a}$ |
| | | | 12 | $0^{a}$ | $0^{a}$ | $3.43^{a}$ | $3.66^{a}$ |
| | | | 15 | $0^{a}$ | $0^{a}$ | $3.98^{a}$ | $3.86^{a}$ |

0 d

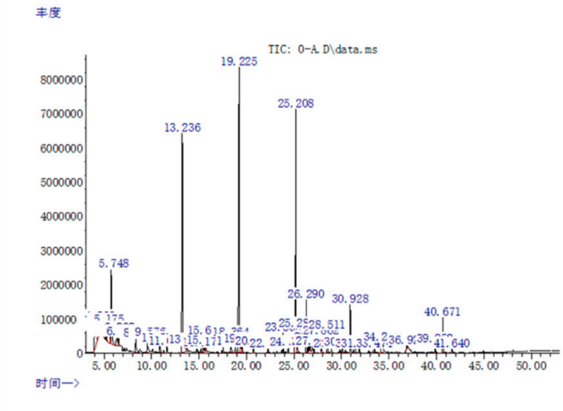


3 d

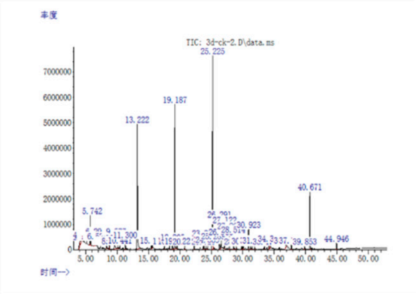


CK

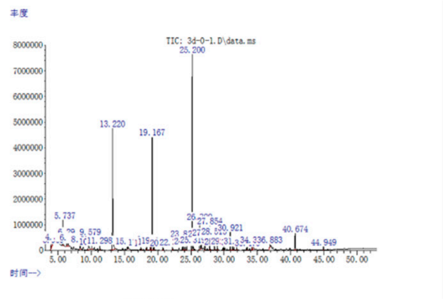


HEC

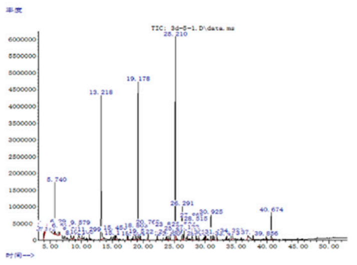


HEC-5%SRBP

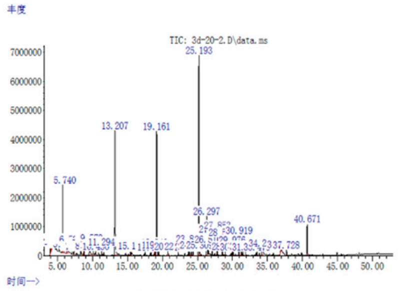


HEC-20%SRBP

6 d

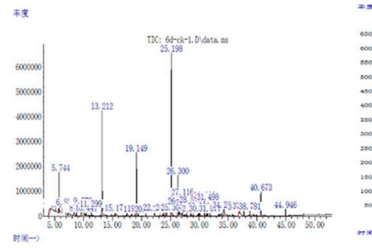

CK

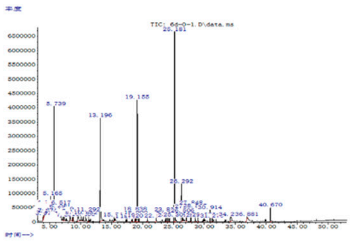

HEC

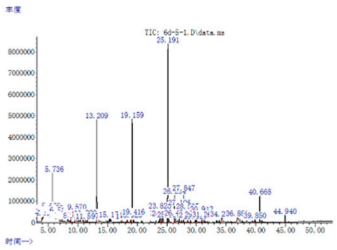

HEC-5%SRBP

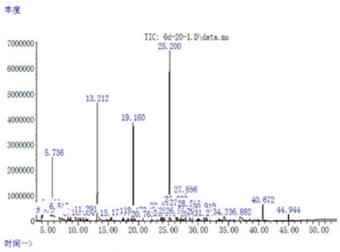

HEC-20%SRBP

9 d

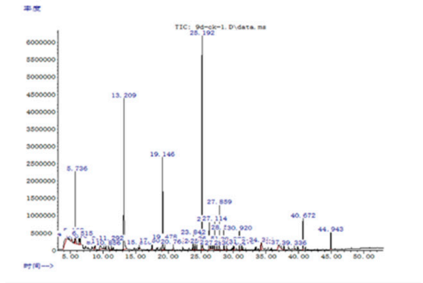

CK

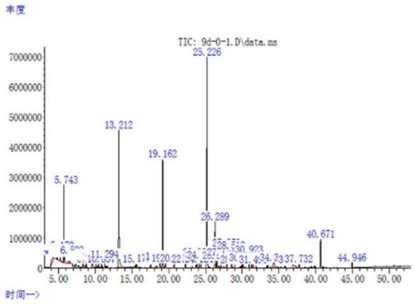

HEC

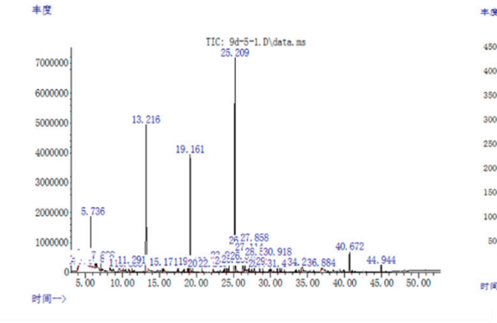

HEC-5%SRBP

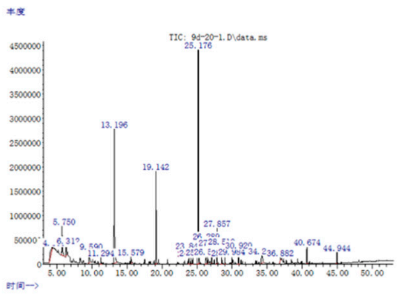

HEC-20%SRBP

12 d

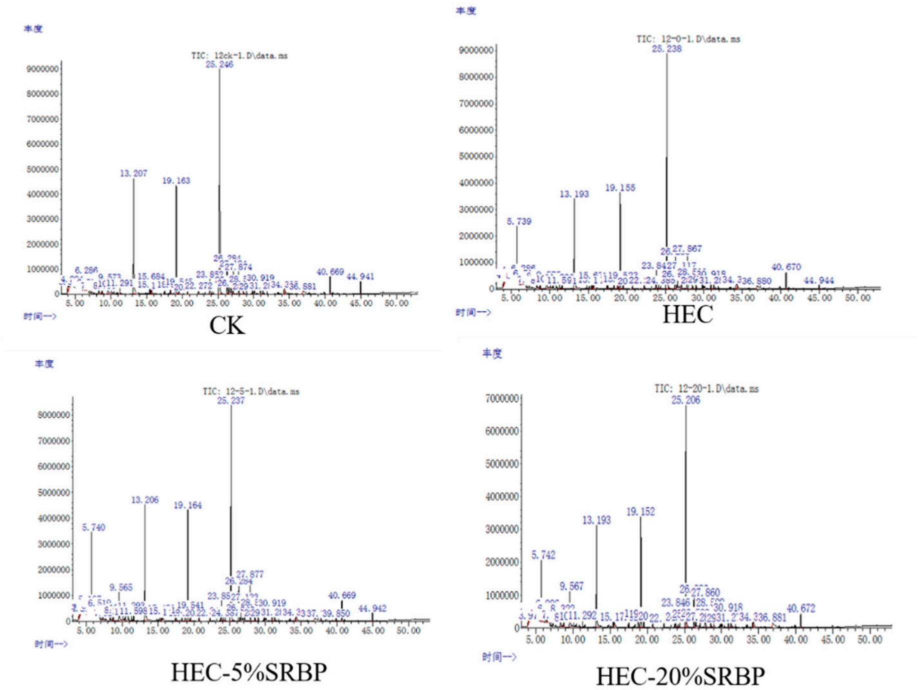


Figure S1. Chromatograms of volatiles substances at different times for different treatments

Table S3. The retention time of n-alkanes

| number | Retention time (min) | Match item name | Molecular weight (amu) | CAS | Spectral library |
|---|---|---|---|---|---|
| 4 | 3.97 | Pentane | 72.094 | 000109-66-0 | D:\GC-MS\GCMS Chemstation\ Raw data for testing \NIST08.L |
| 7 | 4.373 | Hexane | 86.11 | 000110-54-3 | D:\GC-MS\GCMS Chemstation\ Raw data for testing \NIST08.L |
| 21 | 7.432 | Nonane | 128.157 | 000111-84-2 | D:\GC-MS\GCMS Chemstation\ Raw data for testing \NIST08.L |
| 25 | 10.245 | Decane | 142.172 | 000124-18-5 | D:\GC-MS\GCMS Chemstation\ Raw data for testing \NIST08.L |
| 27 | 14.021 | Undecane | 156.188 | 001120-21-4 | D:\GC-MS\GCMS Chemstation\ Raw data for testing \NIST08.L |
| 28 | 18.374 | Dodecane | 170.203 | 000112-40-3 | D:\GC-MS\GCMS Chemstation\ Raw data for testing \NIST08.L |
| 30 | 22.849 | Tridecane | 184.219 | 000629-50-5 | D:\GC-MS\GCMS Chemstation\ Raw data for testing \NIST08.L |
| 32 | 26.748 | Tetradecane | 198.235 | 000629-59-4 | D:\GC-MS\GCMS Chemstation\ Raw data for testing \NIST08.L |
| 36 | 30.025 | Pentadecane | 212.25 | 000629-62-9 | D:\GC-MS\GCMS Chemstation\ Raw data for testing \NIST08.L |
| 42 | 32.897 | Hexadecane | 226.266 | 000544-76-3 | D:\GC-MS\GCMS Chemstation\ Raw data for testing \NIST08.L |
| 49 | 35.483 | Heptadecane | 240.282 | 000629-78-7 | D:\GC-MS\GCMS Chemstation\ Raw data for testing \NIST08.L |

| | | | | | |
|---|---|---|---|---|---|
| 54 | 37.86 | Octadecane | 254.297 | 000593-45-3 | D:\GC-MS\GCMS Chemstation\ Raw data for testing \NIST08.L |
| 57 | 40.08 | Nonadecane | 268.313 | 000629-92-5 | D:\GC-MS\GCMS Chemstation\ Raw data for testing \NIST08.L |
| 63 | 42.165 | Eicosane | 282.329 | 000112-95-8 | D:\GC-MS\GCMS Chemstation\ Raw data for testing \NIST08.L |
